# Letter from Berlin

**Published:** 1891-11

**Authors:** T. M. McIntosh

**Affiliations:** Thomasville, Ga.; Berlin


### LETTER FROM BERLIN.

BERLIN, August, 1891.

*Editors Atlanta Medical and Surgical Journal:*

After leaving London, a short stay of nine days was made in Paris, where there was much of medical interest seen, but chief of which was Apostoli's clinic, who treats all uterine disorders by his method of electricity, and which, in the various uterine hemorrhages, has been of much value in his practice, and of other physicians also, and should, in many of this class of cases, be used before a resort to surgery. The eminent surgeon, Pian, made a vaginal hysterectomy and removed a uterine fibroid and restrained the hemorrhage by the use of hæmostatic forceps, which were left in the vagina about two days; operated for an aneurism of the thyroid artery by placing a forceps on either side of the aneurism, which were removed in forty-eight hours ; amputated at the hip joint and compressed the femoral artery with a forceps also, which he removed in seventy-two hours. This is his method: He never uses silk or catgut to restrain hemorrhage—always the forceps. The wound is dressed in the usual aseptic manner, covering the forceps with gauze and cotton bandage.

Berlin was reached very early in June. From that time until now, the surgical clinics of all the eminent specialists have been visited daily, some by payment of the price required, and some by the courtesy of personal invitation. From both sources one can have abundant observation and instruction. The material is abundant and absolutely in the hands of the surgeon, and students can examine and reëxamine. The patient takes it as a matter of course. And this applies as well to diseases of women as other diseases. In America, the women are somewhat averse to public examination and treatment, but here it is not true, and for this reason the opportunities for study in this department are surpassingly good. No such thing as a sheet to cover a woman during an examination is thought of, and the universal position of the examiner is between the legs of the patient, and is better than the usual method in America, at the side.

The leading men here in surgical gynecology are Olshausen, of the University, the successor of Schroeder; Gusserow, of the Charité, and Martin, with his private clinic.

As abdominal surgery is the department in which the greatest advance has been made in the past few years, and the principles which underlie it are those which will apply to all surgery, and as its technique is of importance, one cannot give more interesting matter than to describe in a measure these men and their methods.

Martin, as every one knows by reputation, is a very large, fat man, blind of one eye by some casual injury. He has a heavy, coarse face, which is a true reflex of the man. He is not personally popular in Berlin, and here is not so famous as either Olshausen or Gusserow. He is also not popular with his students. He gives private courses of one month, for which one pays a very reasonable sum, and when one takes this, letters of introduction are not needed, and mine was not presented, so, as I received no courtesies from him, having paid for what I saw, these remarks are not improper. He and his female assistant are surely a wonderful pair. He operates every day, and one can stand for hours and see kolporophies, and perineorophies, and uterine curettements, and cervical amputations made in rapid succession. No man can use a needle and catgut like Martin. He is a wonderfully rapid operator. All of his operations, except laparotomies, are made in the ordinary operating room. The patient is chloroformed on the table. She is then brought well down to the end, with the nates projecting a little beyond the edge; the vulva is shaved by his woman assistant, the parts and surroundings well washed with soap and water, and then with a solution of bichloride, the vagina being cleansed by the fingers thrust in and the sides separated, and water copiously poured in, the fingers meanwhile rubbing and scouring every part of it. An assistant takes each a leg, which is carried well back and put into a rest, which brings the patient into an exaggerated lithotomy position. Martin, with a long rubber apron on, takes a seat in front, puts the end of the apron in a large vessel under the end of the table, puts his short and wide-bladed speculum in the vagina. If he is making a kolporophy, with a long bullet forceps he draws down the uterus, gives this to an assistant, who holds it vertically between the legs, and in the same grasp the straight point of an irrigator, from a vessel holding sterilized water. The lowest point of the proposed removal of mucous membrane is grasped by another forceps, given to assistant No. 2, who holds it vertically downward. The free hand of each assistant holds the side retractors that are put in the vagina. He cuts out rapidly, and without attention to bleeding, the tissue, and with a curved needle threaded with catgut makes a stitch at the apex of the incision, ties it, leaving the free end long, which he gives to the assistant, who now removes the forceps, and makes traction with this catgut. He then proceeds to approximate the sides of the cut by the continuous buried suture, thus building a support from the vaginal tissue, which may require more than one layer of the buried suture before the mucous cut surfaces are sufficiently approximated to bring them together with but little traction. If he is making an operation for rupture of the perineum, he fixes the four points of the parts to be vivified with as many bullet forceps, which his assistants hold, dissects rapidly, and builds from the bottom by the buried sutures, as in the kolporophy, so he does not use the long perineum needle for the usual deep and interrupted suture. If the rupture is complete, he will put in one or two deep silver wire sutures. He will make a vaginal hysterectomy in from thirty to forty-five minutes, while all of the other operators do so in from fifty to seventy-five minutes. With his short, wide speculum pressing the perineum, the retractors on either side of the vagina all being well drawn up, he fastens the bullet forceps through both lips of the uterus and pulls this well out. He then cuts into the posterior cul-de-sac, passes in his finger, and with the point of this well against the body of the uterus pressing out toward the vagina, he passes catgut with a sharp-pointed curved needle (his finger acting as a guide to the point of the needle and its depth) and ties every particle of tissue with many different ligatures in advance of his incisions, which he makes with curved scissors. So soon as he can, he draws the fundus uteri without the vagina, inverting completely the uterus, and makes the separation from the bladder last. The ligatures are cut short, the vagina is filled with iodoform gauze, and the operation is complete. The hemorrhage is almost nothing, and what there may be is washed away by the slow stream of the irrigator. He has no sponges or gauze to remove blood during his operations.

His ovariotomies are made in a special room, as is the case in every clinic in Germany. This room is heated to about seventy degrees or more. Every spectator strips himself of coat and vest, but puts on no white coat. He evidently has a pride in the rapidity of his operations, for as soon as he has passed the last stitch he asks an assistant, who takes his case history, how long the operation has been. He sits directly between the legs of his patient, which hang on either side of him, and makes a long cut from the umbilicus to near the pubic bone, and pays no attention at all to the parietal blood vessels. When the peritoneum is reached, he grasps a bit of it with two sharp pincettes, an assistant takes one, and between them it is cut through with the scissors, two fingers are inserted, and with these as a guide he cuts rapidly the entire wound. If the tumor is cystic, he cuts a pus opening with a bistoury and pulls the incision over the side of the abdomen until it is well emptied, and takes no special pains to prevent the contents from getting into the peritoneal cavity. The intestines are held out of the field of operation by an assistant with a large, flat sponge in the peritoneal cavity which has been sterilized and then boiled for four hours in olive oil. The bleeding from adhesions which he tears with the hand are tied with catgut. The pedicle is fixed by catgut sutures, several of which he will pass, owing to the size of the pedicle, and each tied separately and in sections, and then the ends of the central one passed entirely around and tied. The pedicle is dropped into the cavity, which is then cleansed with sponges from sterilized water. No antiseptic or antiseptic purified instruments go into the cavity. In *closing* the abdominal wound, he uses stout *silk*, by using five, six or seven deep interrupted sutures, the entrance and exit of which are an inch or more from the cut surface, and the course almost perpendicular until near the peritoneum, through which it passes quite near the cut edge. These are tied, and the skin edges brought together by the *continuous* CATGUT suture. A little iodoform and iodoform gauze are put on the wound, and over these some cotton batting is bandaged very securely.

The time required to make a laparotomy is from eight to forty-five minutes. This last was a very large uterine fibroid. The incision was made beyond the umbilicus, and as there was free, uncontrollable hemorrhage from the vessels of the tumor itself, he cut this off near the pedicle, and then with forceps and catgut entirely around the pedicle he restrained the bleeding, and dissected out the remaining portion of the tumor in the usual way. The body of the uterus was removed and an opening made in the anterior cul-de-sac, through which the free ends of the numerous ligatures used were brought, to act as a drain through the vagina, which was well filled with iodoform gauze.

In his clinic there was a young woman who had had an ovary removed in 1889 and the other in 1890, for excessive uterine bleeding, and she came now for a continuance of this. The uterus will be curetted, and by the repeated injection of strong solutions of zinc chloride an effort will be made to close the uterine cavity by granulation. Here is a case where surgery failed to relieve a uterine hemorrhage, and just the class of cases in which Apostoli, and at present Thomas Keith, who uses the Apostoli method, claim that they obtain such markedly good results from electricity.

Olshausen is the successor of the famous Schroeder, and is universally, personally and professionally esteemed. He is in appearance and reality a gentleman.

He does not operate so rapidly as Martin, but more carefully The antisepsis of each is the same, with perhaps some more care on the part of Olshausen. In his vaginal and uterine operations, he uses never a sponge, but gauze sterilized by steam, and once used is thrown away. This he also uses in the peritoneum in his laparotomies. Catgut is this universal suture and ligature. He builds from the bottom with the buried suture in perineal and kolporophy operations.

Hysterectomies he makes by incisions entirely around the cervix and carefully cutting with scissors and dissecting with finger, and usually separates the tissue equally all around, and enters the peritoneal cavity first, sometimes anteriorly and sometimes posteriorly. He passes his hæmostatic sutures with a long, curved aneurismal needle, and seeks soon to tie the two laterel uterine blood vessels with the round ligaments. He uses less sutures and less cutting and more turning than Martin, but his operation is not so bloodless and rapid. His incision through the abdominal wall in laparotomies is carefully made and the hæmorrhage stopped before the peritoneum is opened. Bleeding from torn adhesions is tied by catgut, and the pedicles are treated in the usual German way—ligation in sections. The peritoneum is sewed separately, and the abdominal tissue also, from the bottom, with the buried suture; the skin last.

One day his assistant, Dr. Winter, made a vaginal hysterectomy. He divided tissue before he had passed the preceding hæmostatic ligature, from which there was free bleeding. From repeated attempts he failed to tie this part, as it was deep in the pelvis and against its wall, though he could catch it with the forceps. He tamponed, toiled half an hour, opened the abdomen secured the vessel, and the patiant got well. Here was a case where he should have let the forceps remain, after the method of Pian.

In company with a prominent gynecologist of Chicago, a stay of three days was made at Leopold's Clinic, of Dresden, in response to an invitation in reply to a letter asking if we might visit his hospital. He is quite a young man, being only forty-five years of age, and is exceedingly courteous to visiting physicians. He has made, perhaps, more Cæsarean sections and with better results than any other operator, but even his results are not so good, either as to the number of women and children saved, as by the premature induction of labor and the use of an incubator in which a five months fœtus will live and thrive. Leopold heads the list in the number of his vaginal hysterectomies, having made very near two hundred, with a mortality of less than 5 per cent. Eighty of these were made for carcinoma, forty-five of which are living two years and longer after operation, the longest being seven years. It is presumed in carcinoma cases that if there is no return in two years it is cured.

His entire hospital is a model of cleanliness and antisepsis. He uses great quantities of bichloride. In the lying-in wards a vaginal examination is never made, as the position of the child is diagnosed by external touch. We saw him make a laparo-hysterectomy for carcinoma. The patient was placed in Trendelenberg's position (the hips elevated at an angle of perhaps forty-five degrees). The abdominal incisions were made boldly and with but little care as to the bleeding, the uterus with surrounding carcinomatous tissue isolated, in doing which it was necessary to dissect it from the left ureter, and large attachments to the rectum also. When this was complete, an opening was made through the vaginal vault and iodoform gauze stuffed in, the abdominal wound closed, the patient's hips lowered, and the operation completed by removing the organ through the vagina, some of the wall of which was cut away, into which the disease had extended.

Leopold makes all of his laparotomies with the hips of the patient elevated, and one is pleased at the perfect view one can have of the pelvic viscera. The intestines fall down toward the thorax and require no holding and do not fall out.

I have seen Olshausen remove suppurating tubes that were firmly adherent *in situ* with this position of patient, and it seems to me that it is a comfortable one for the operator. Certainly the view is perfect.

Leopold uses nothing but silk in his operations, and does not use the buried suture in his kolporophies and perineorrophies, and because the stitches have to be removed, is not so good as catgut.

He has a new operation for obstinate retroversion of the urethra, the first of which we saw him make, which he hopes to be able to substitute in many cases for ventral fixation. Briefly, it consists in encircling the entire uterine cervix with an incision well through the mucous membrane, and then from that one, and at right angles to it, another on either side of the cervix its entire length, with the finger chiefly dissecting the mucous membrane anterior and posterior, well up to the body of the uterus, the extent of the lateral incision.

Where the lateral cervical incision begins a slant suture is inserted and brought out, carried up to the highest point of dissection, inserted and passed buried to the opposite side, in front of cervix, brought out again, and then carried down and passed through tissue on the opposite side to where it was first inserted. This parallelogram of ligatures, drawn upon, brings the upper anterior part of its dissected surface down to the incision over the mouth of the cervix, and as the cervical edge of the flap is held also by the suture, it folds this on itself and bulges it, so to speak, forward and shortens the anterior surface of uterine mucous tissue. This pulls upon the fundus and draws it forward and stretches the posterior dissected surface, which is left to heal by granulation, and acts as a posterior stay to the womb.

All in all, Leopold is perhaps the best operator I have seen. He is rapid, sure, scrupulously clean, and makes the prettiest and neatest incisions and the most perfect approximation and stitching.

My observations since in Europe have convinced me that for all abdominal operations catgut suture and ligature is better than silk, because it is absorbed and cannot act as a foreign body, and because silk is not absorbed and may be a foreign body. The objection that catgut is unreliable by reason of its short life or absorption I am convinced is not well founded, and this is based first, upon Pian's use and removal of the hæmostatic, which proves that a firm clot is found in the largest artery in three days, and because of the almost universal use of catgut here and no accidents occurring.

However, as catgut is expensive, and the rapid formation of thrombus is based upon a thoroughly aseptic wound, it is perhaps best that the average operator, in all other than abdominal surgery, should yet use silk, because the asepsis, not being so uniformly possible to the physician who does all work, a firm thrombus *might* not be found before the catgut is absorbed, and the silk can come away as a foreign body later and only prolong the time of cure. Abdominal wounds *must* be right or the patient is dead. Other wounds *can* be septic and the patient is only longer in bed perhaps.

I meet here such distinguished men as Wm. Goodell, of Philadelphia, seeing these men and their methods.

In future I shall hope to write of Hirschberg and Schwiegger, the eminent oculists, whose clinics I have visited daily for two months, as they were at different hours ; also of Von Bergmann and his surgery, perhaps.

I shall go to Vienna the last of September.

T. M. McINTOSH.

*Of Thomasville, Ga.*

